# Bilateral complex lactating adenosis: Clinical presentation and management in Sudan

**DOI:** 10.1002/ccr3.5213

**Published:** 2021-12-13

**Authors:** Hatoun Dafaalla, Hussam S. Khougali, Yussra Malassi

**Affiliations:** ^1^ Department of General Surgery Wad‐Medani Teaching Hospital Wad‐Medani Sudan; ^2^ Western Sussex Hospitals NHS Foundation Trust Worthing UK; ^3^ Department of Research and Methodology Ibn‐Seina Hospital Khartoum Sudan

**Keywords:** breast cancer, fibroadenoma, lactating adenosis

## Abstract

Lactating adenosis is a benign breast neoplasm usually seen during pregnancy and lactation. We present a rare case of bilateral complex benign lactating adenosis in 19‐year‐old female patient presented with lactating adenosis that mimics cancer growth after giving birth. Histopathology confirmed diagnosis. Simple mastectomy was the treatment of choice.

## Graphical abstract

Menstruation and pregnancy in teen females can be followed by an aggressive type of lactating adenosis. Little is known about management of lactating adenosis. A simple mastectomy can be the treatment of choice for such complex lactating adenosis.

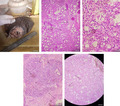

## 1 BACKGROUND

Lactating adenomas are benign tumors of the breast and are most commonly found during pregnancy and lactation.^1^ They are mostly small (<3 cm) and slow growing, although some cases of larger lactating adenomas have been reported in the literature as rare entities.^2^ This is a very complex case of benign lactating adenosis to be reported in Sudan. However, little is known about lactating adenosis and other benign breast tumors. The origin of lactating adenomas is disputed. However, it is believed to be either de novo or a modification of a preexisting lesion such as fibroadenoma or tubular adenoma, reflecting the changes resulting from the physiological state of pregnancy induced mainly by hormones.^1^, ^3^, ^4^ Nevertheless, the exact physiological mechanism of the role of hormones in such growth remains controversial; some studies have linked the rapid growth with the concentration of prolactin.^5^ The heterogeneity in the glandular component of lactating adenomas may be encountered; hence, the difficulty in distinguishing between lactating adenosis, benign phyllodes, and adenocarcinoma of the breast may arise.^6^ Overall, lactating adenosis is known to have benign behavior and does not carry a high risk of malignant transformation.^7^, ^8^ Lactating adenosis has got very little interest in Sudanese literature; all focus is on the malignant neoplasm of the breast; however, the complex cases of benign breast neoplasm usually add some clinical complexities and management difficulties for many Sudanese clinicians, which may be associated with negative impact on some patients. This article aims to discuss a case of bilateral complex lactating adenosis that mimics cancer features in young Sudanese female as well as review in literature and analyses the clinical practice of such kinds of complex benign neoplasms of the breast.

## 2 CASE PRESENTATION

A 19‐year‐old female from east Sudan presented 3 months after giving birth of her first healthy baby with huge multiple bilateral breast masses. She was very anxious about this bilateral tumor growth in her both breasts. This tumor started to grow 2 years prior to the presentation (age 17). Initially, the masses appeared in the right breast and shortly involved the left one. Lumps showed a gradual course initially, but a dramatic rapid growth was noticed during pregnancy. There was severe discomfort especially in the left breast, due to rapid growth and inflammation. There was no past history of breast trauma or family history of breast cancer. The patient started to menstruate at the age of 15 years, without reporting any abnormal events in her breasts. She was of good internal and external hygiene; no history of smoking, drinking, or any bad habits; and no allergies. Both breasts had multiple lumps; the right breast mass was measuring about 7 cm × 7 cm, hard and nodular with no skin changes or evidence of deep structure involvement by clinical palpation. The largest left breast mass was about 7 cm × 5 cm with other smaller ones occupying all four quadrants. There was large area of skin changes, which surrounds the left large lump with a large irregular inflamed ulcer below and lateral to the left nipple with no elevated edges. Both breasts were lactating, and milk discharge from the nipple was observed with normal color.

**FIGURE 1:**
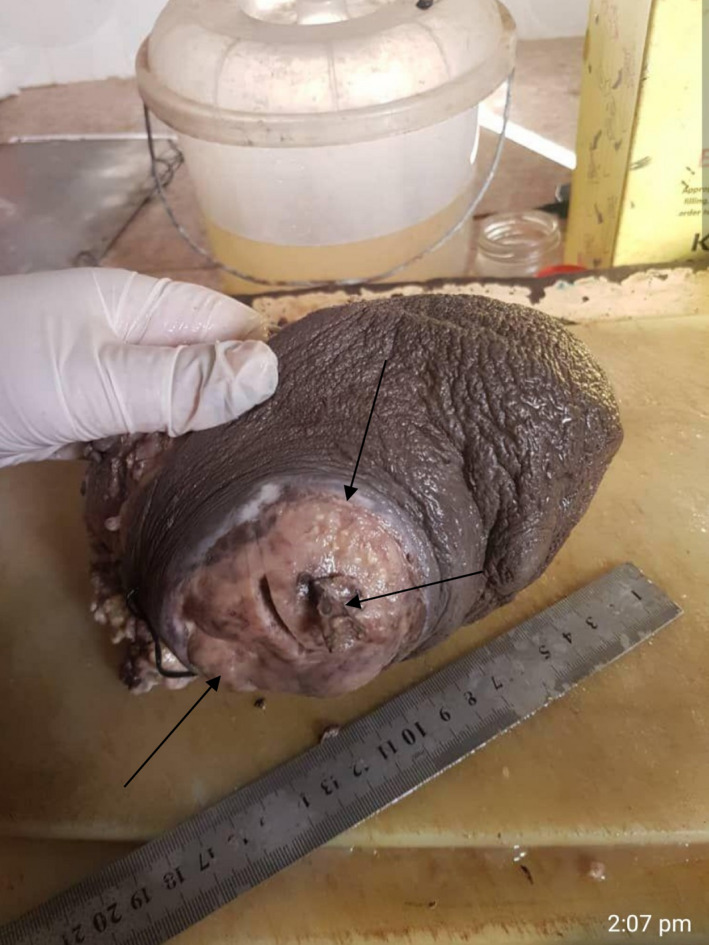
Growth section of the left breast mass after simple mastectomy, showing ulcerated huge lactating adenoma not responding to conservative management. Arrows show the size of the tumor and ulcer; nipple is completely invaded by ulcer and inflammation

**FIGURE 2:**
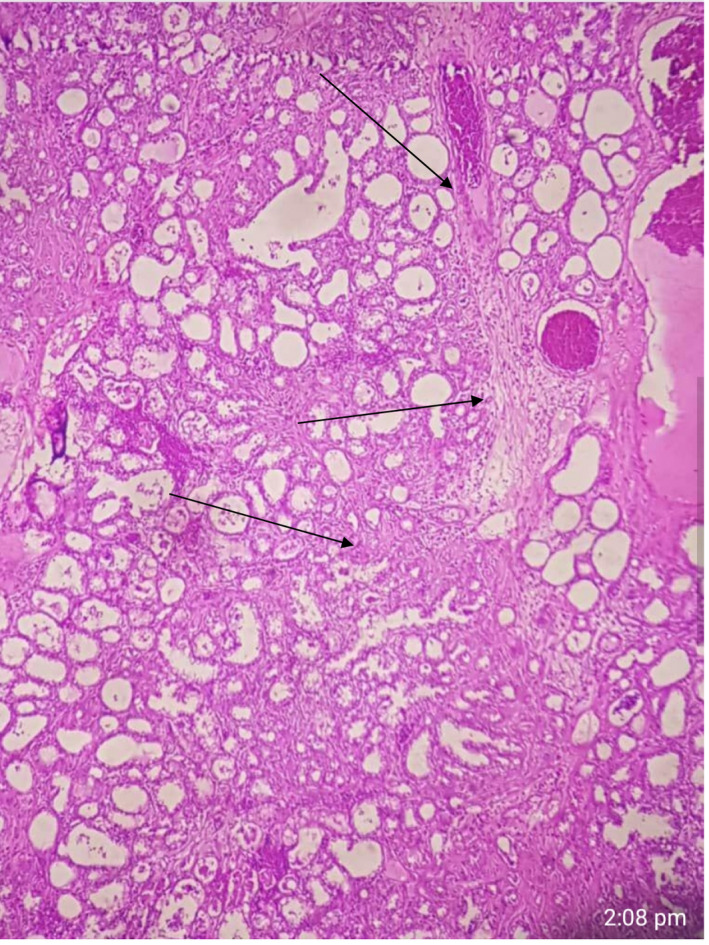
Left breast medium power view blebbing and secretory changes in complex glandular architecture

**FIGURE 3:**
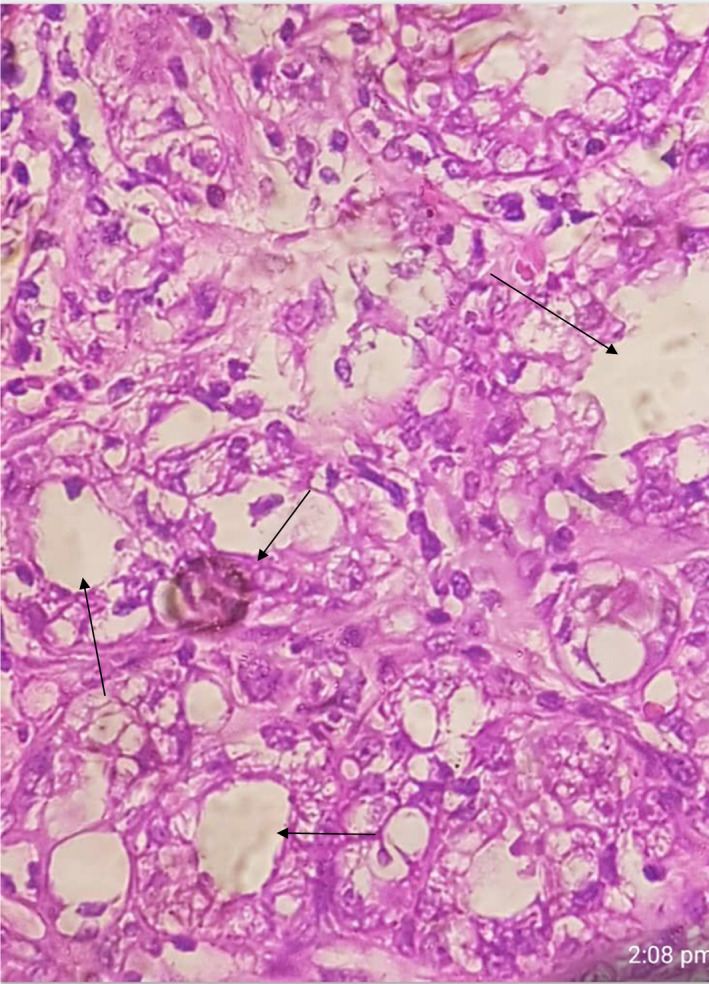
Left breast high power view, bland cytological features with cytoplasmic vacculations and secretory changes

**FIGURE 4:**
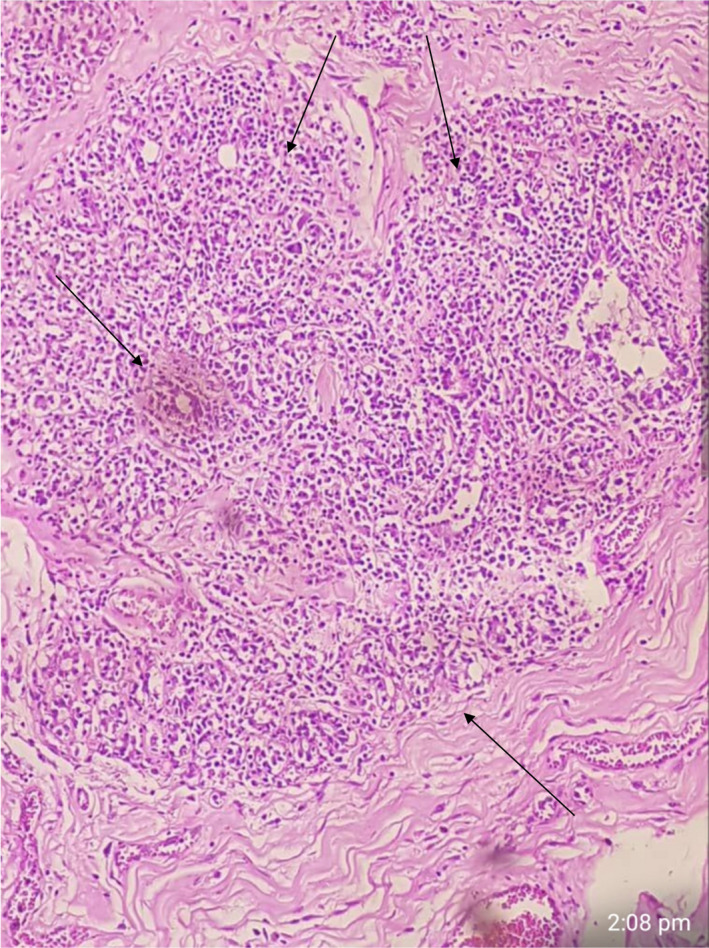
Right breast medium power view shows an enlarged lobule filled with glands

**FIGURE 5:**
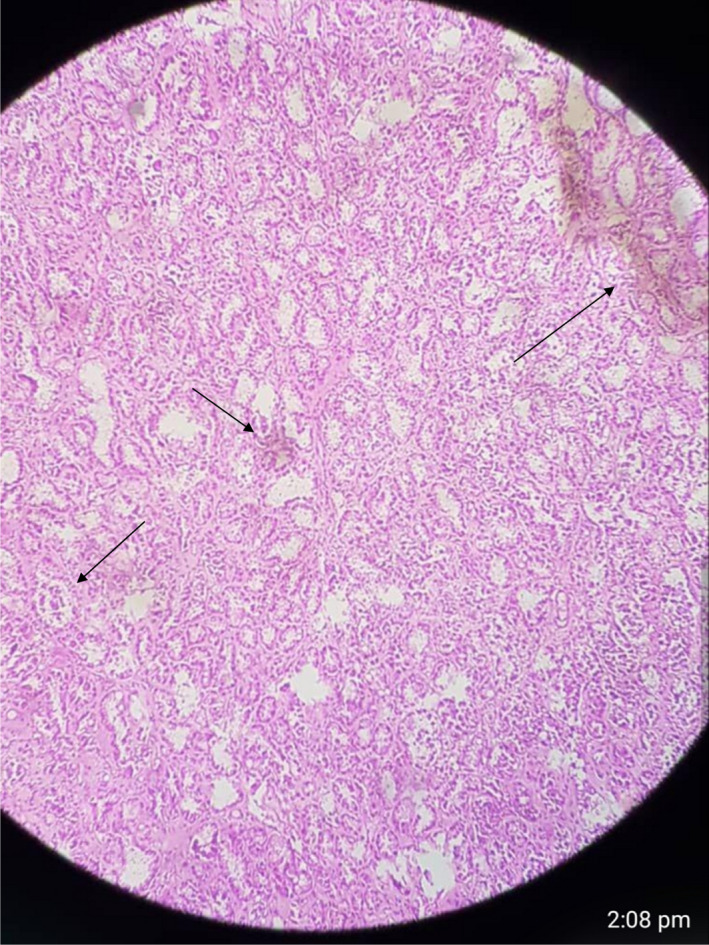
Right breast low power view shows a neoplasm with densely packed glands

Ultrasound report of the left breast showed multiple bilateral echogenic breast enlargements; the largest one was about 7 cm × 7.55 cm. Also, there was a 7 cm × 4 cm well‐defined, turbid, and cystic collection noted beneath the ulcerated area in the left breast (Figure 1). Dilated right breast mammary ducts were noticed. The nipple and overlying skin were intact. Multiple enlarged bilateral axillary lymph nodes (LNs) were identified. The cytology report confirmed area of hyalinization with no malignant changes. Patient completed 6 months of conservative management (bromocriptine and antibiotics) locally without any signs of improvement. The size of the tumor steadily growing, inflammation, ulceration, and LN enlargement were getting worse with time. Consequently, left simple mastectomy had been done based on failure of conservative management and infiltration of other quadrants in the left breast with very huge ulcerated tumor. Active surveillance with watchful wait was management plan for the right breast mass. The excised specimen's histopathological report revealed macroscopically an exophytic growth measuring 7 cm × 6 cm lateral to normal looking nipple with skin ulceration. Cross sectioning of the specimen showed no breast tissue, only lobulated firm mass with central cyst behind the nipple measuring 10 cm × 7 cm × 5 cm in diameter. Microscopically, sections showed enlarged lobules, with complex glands composed of inner actively secreting epithelial cells with vacuolated cytoplasm and apical cytoplasmic blebs and outer myoepithelial layer (Figures 2 and 3). There was no cytological atypia, and stroma was scanty. There were two reactive isolated axillary LNs identified during histology; the right breast showed the same histological features (Figures 4 and 5). P63 and S100 were positive. The immunohistochemistry confirmed a diffuse tumefactive lactating adenosis with very complicated histological picture. Eventually, 6‐month follow‐up was arranged, and overall prognosis after surgery was satisfied.

## 3 DISCUSSION

Lactating adenomas are benign tumors of the breast and are most commonly found during pregnancy and lactation. This is a very complex case of benign lactating adenosis reported in Sudan. However, fibroadenoma is one of the commonest benign breast tumors in young females in Sudan, but little is known about lactating adenosis and other benign breast tumors.

Scholars such as O'Hara and Page^9^ believe that lactating adenomas have precursors, including fibroadenoma and tubular adenomas. These lesions then undergo secretory changes associated with physiological state of pregnancy. These give rise to controversies that may belong to different nature of the growth, which is influenced by the effect of hormones on the breast both during pregnancy and lactation period.^10^ About 3% of breast cancers are diagnosed during pregnancy; hence, possibility of coexistence of benign and malignant lesions should not be neglected.^11^, ^12^

Many cases of lactating adenoma that have been mentioned in the literature were of atypical presentation. Some of them are even with an aggressive presentation. Though lactating adenomas are rendered to benign behavior, the literature has mentioned the coexistence of benign and malignant tumors in the same patient,^9^, ^12^, ^13^ others in the same anatomical site.^14^ Hence, caution must be taken while dealing with lactating adenoma when the patient comes with atypical presentation or with signs and symptoms suspicious of carcinoma as in this case, which presented with features that mimic inflammatory breast carcinoma.

The relation between the effect of females' hormones and breast tumors is not well understood, and many studies have subjugated this complex relation to intense investigations. Gill et al^5^ concluded in their study that a high prolactin concentration has a positive correlation to female breast tumors. However, the exact mechanism remains very sophisticated.^15^ In our patient, the complicated growth picture, enlarged LN, and the presence of ulceration may be attributed to the hormonal changes during pregnancy and lactation, especially the growth picture in this case seems against the classical histology of lactating adenosis that appears as well‐circumscribed, lobulated, solitary or multiple, and gray with areas of necrosis; microscopically, they are seen as cuboidal cells with actively secreting, closely packed glands.^16^

Triple assessment is very recommended in such cases in order to differentiate between adenoma and carcinoma of the breast,^16^, ^17^ but even though with such complex growth, the distinguishable point from cancer clinically remains very difficult, and only histology can give clear differentiation. The detection of positive P63 in the histopathology has been linked to underlying ductal carcinoma in situ and some cases of invasive ductal carcinoma, and this adds sounds of doubt for future behavior of this benign tumor^17^; a simple mastectomy is the safest option for such huge ulcerated lactating adenoma, which is resistant to conservative management.

In conclusion, menstruation, pregnancy, and lactation in teen females can be followed by an aggressive type of lactating adenosis with a complex macro‐histological picture that mimics cancer growth. Moreover, under the influences of puerperal hormones, bilateral breast involvement can be inevitable. The complex growth of giant lactating adenosis can be missed as cancer even with usual triple assessment, and the immunohistochemistry is the mainstay of differentiation. A simple mastectomy can be a very effective treatment for such complex lactating adenosis with no response to conservative or medical treatment.

## CONFLICT OF INTEREST

The authors declare no conflict of interest.

## AUTHOR CONTRIBUTIONS

H. D., H. S. K., and Y. M. all have the same contribution in this article: writing, editing, and approval of the manuscript for publication.

## ETHICAL APPROVAL

The authors declare that this article does not involve human participant and/or animal.

## CONSENT

Written informed consent was obtained from the patient to publish this report in accordance with the journal's patient consent policy.

## Data Availability

The data that support the findings of this study are openly available in PubMed and Google Scholar.
